# Supplementary material for: Network Theory Analysis of Allosteric Drug-Rescue Mechanisms in the Tumor Suppressor Protein p53 Y220C Mutant
Source: Int J Mol Sci. 2025 Jul 17;26(14):6884. doi: 10.3390/ijms26146884 (PMC12295982; doi:10.3390/ijms26146884)
Supplement: Supplementary file 1 [file ijms-26-06884-s001.zip › Supplementary_Information_and_Figures.pdf]

Supporting Information and Figures

Benjamin S. Cowan[1] and Kelly M. Thayer[1,2,3*]

[1,2]Department of Computer Science
Wesleyan University
Middletown, CT 06457 USA
[2]Molecular Biophysics Program,
Wesleyan University
Middletown, CT 06457 USA
[2]Department of Chemistry,
Wesleyan University
Middletown, CT 06457 USA

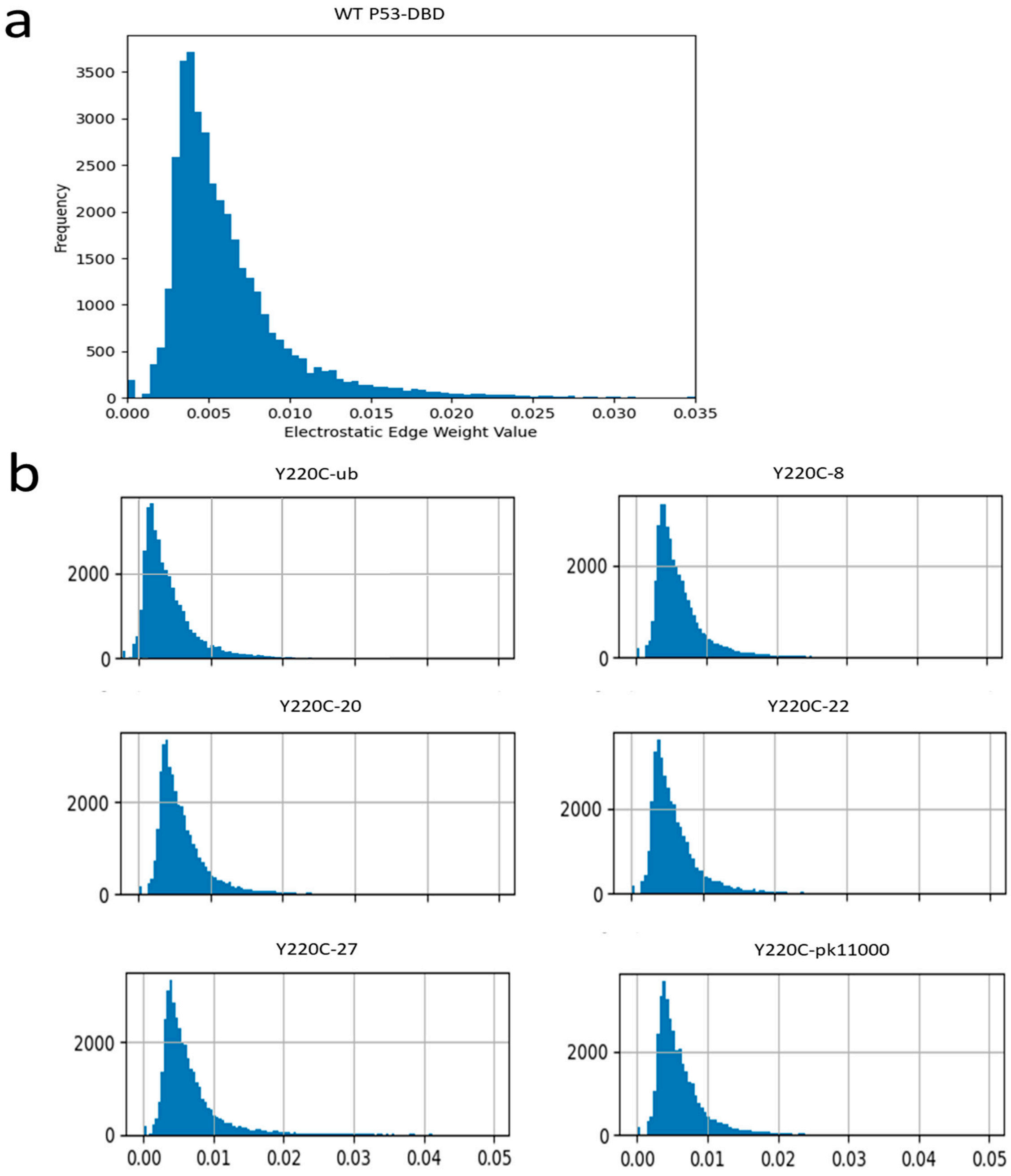

Supplementary Figure S1. Histogram of Normalized Edge weight values for Locally Thresholded and Normalized Electrostatic residue interaction networks for p53-DBD constructs. (a) Histogram edge weight distribution of the normalized 193x193 pairwise electrostatic interaction networks of the wildtype p53-DBD across all 152 trajectory frames. (b) Histogram edge weight distributions of the normalized 193x193 pairwise electrostatic interaction residue networks of the six Y220C mutant p53 systems' DBDs across each of their respective simulation's 152 trajectory frames. X-axis represents locally thresholded and normalized edge weight value with y-axis representing frequency.

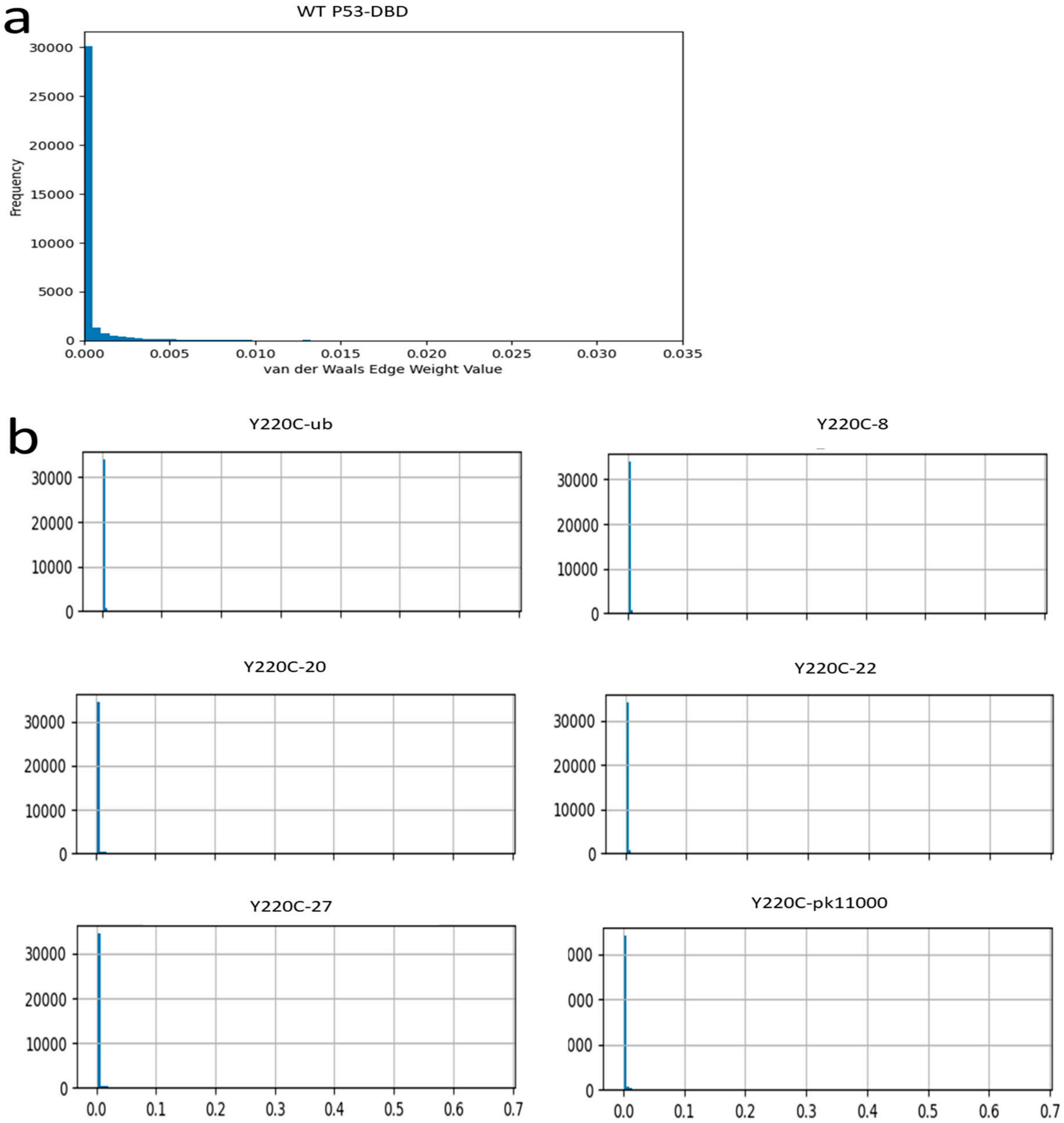

Supplementary Figure S2. Histogram of Normalized Edge weight values for Locally Thresholded and Normalized Van der Waals (VDW) residue interaction networks for p53-DBD constructs. (a) Edge weight distribution for the normalized Van der Waals energetic interaction networks of the wt p53 system across all 152 trajectory frames. (b) Histogram of edge weight distributions for the normalized Van der Waals (VDW) 193x193 pairwise residue energetic interaction networks of the y220c unbound mutant and five y220c-mutant effector drug-bound p53 system DBDs' across all their respective trajectories' 152 frames. Noticeably, the values of these mutant systems' VDW edge wights display a similar distribution to wt p53's VDW edge weight values, approximating at zero. X-axis represents locally thresholded and normalized VDW edge weight value with y-axis representing frequency.

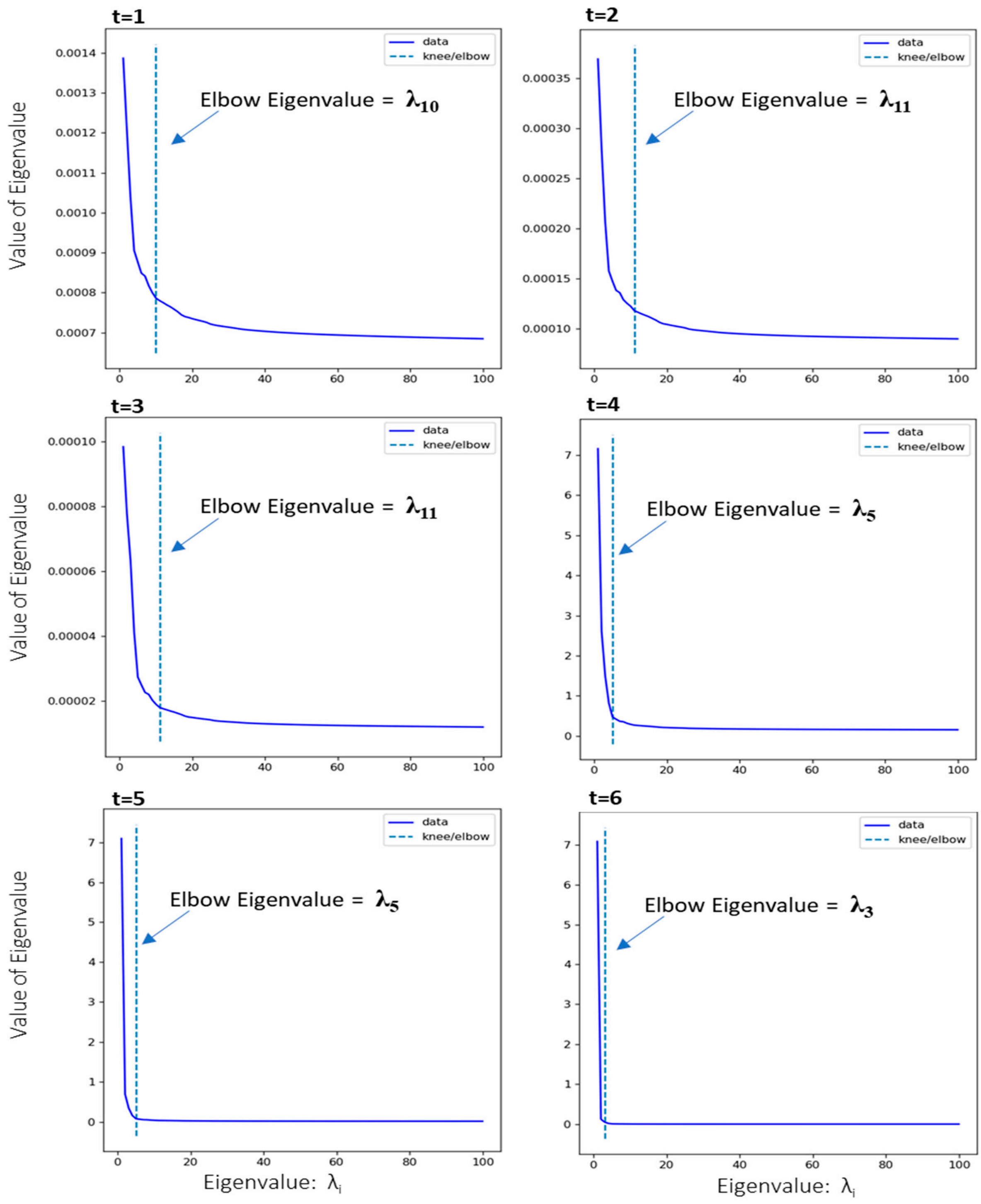

Supplementary Figure S3. Generated scree plots of the first 100 eigenvalues of the mean centered kernel of the wildtype p53 heat kernel for various values of the time parameter, t. X-axis represents eigenvalue/principal component number and y-axis represents its corresponding value. The dotted blue line of each line plot represents the computed needle/elbow point eigenvalue of each plot's data. The elbow point eigenvalue delimitates the necessary number of principle components to describe the majority of variance in the heat kernel in the PC-space.

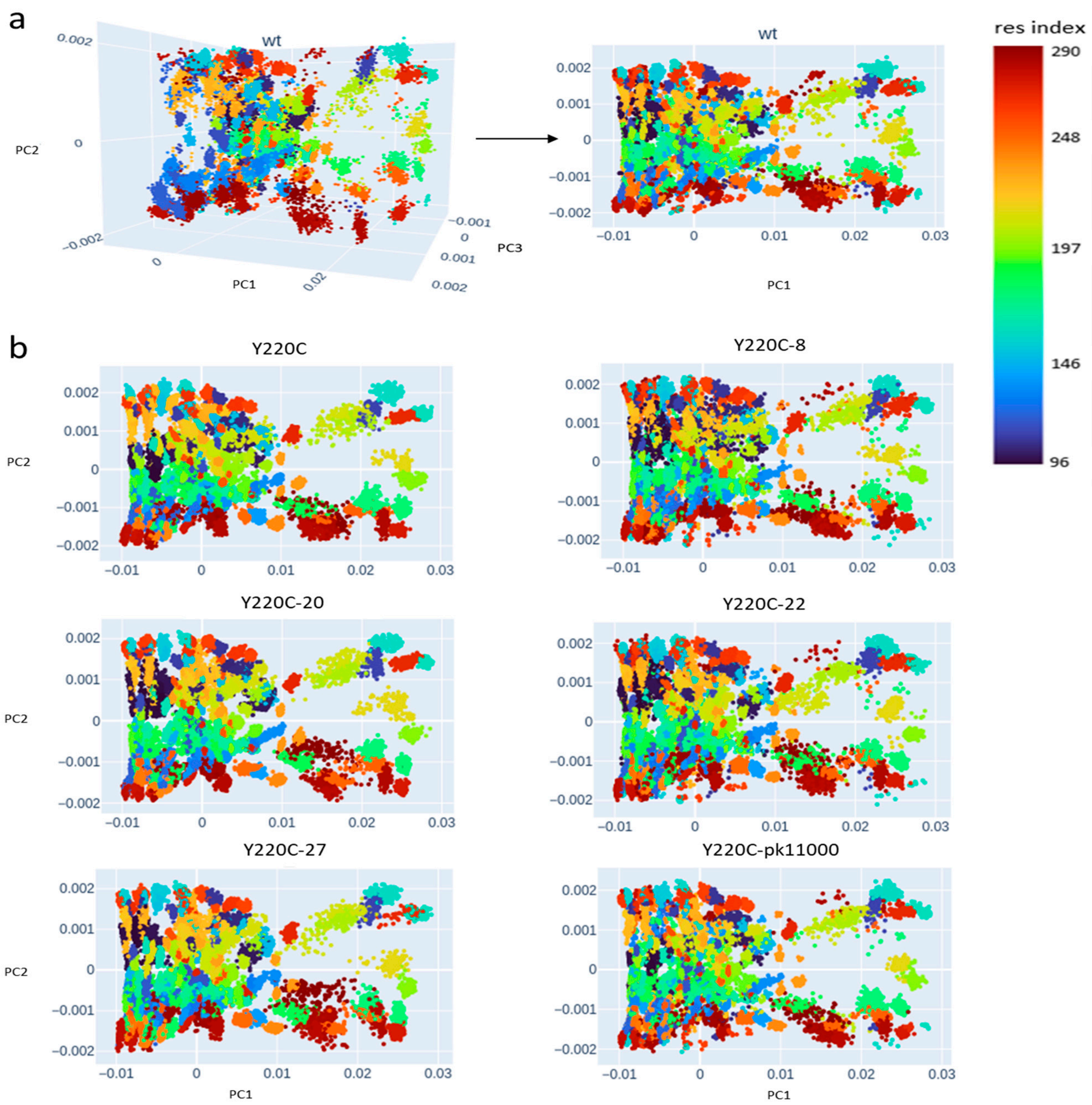

Supplementary Figure S4. Electrostatic heat kernel Principal Component (PC) projections of all 193 residues for each of the seven investigated p53 DBD constructs' 152 sampled frames of simulation. In total there are 152x193 =29336 embedded points in R^3 principal component space for each construct. (a) The projection of the wildtype construct's 152 heat kernels in R^3 across PC1, PC2, and PC3 (left) and R^2 across PC1 and PC2 (right) are displayed. (b) While calculated in R^3 , the projections of the remaining six constructs: y220c, y220c-8, y220c-20, y220c-22, y220c-27, and y220c-pk11000 are only shown across R^2 for clarity. A y220c construct bound to the effector drug compound j is denoted as y220c-j. Color mapping corresponds to the associated residue index each node embedding represents. For each residue in each construct's associated plot, there are 152 associated heat kernel node embedding points in the projection space.

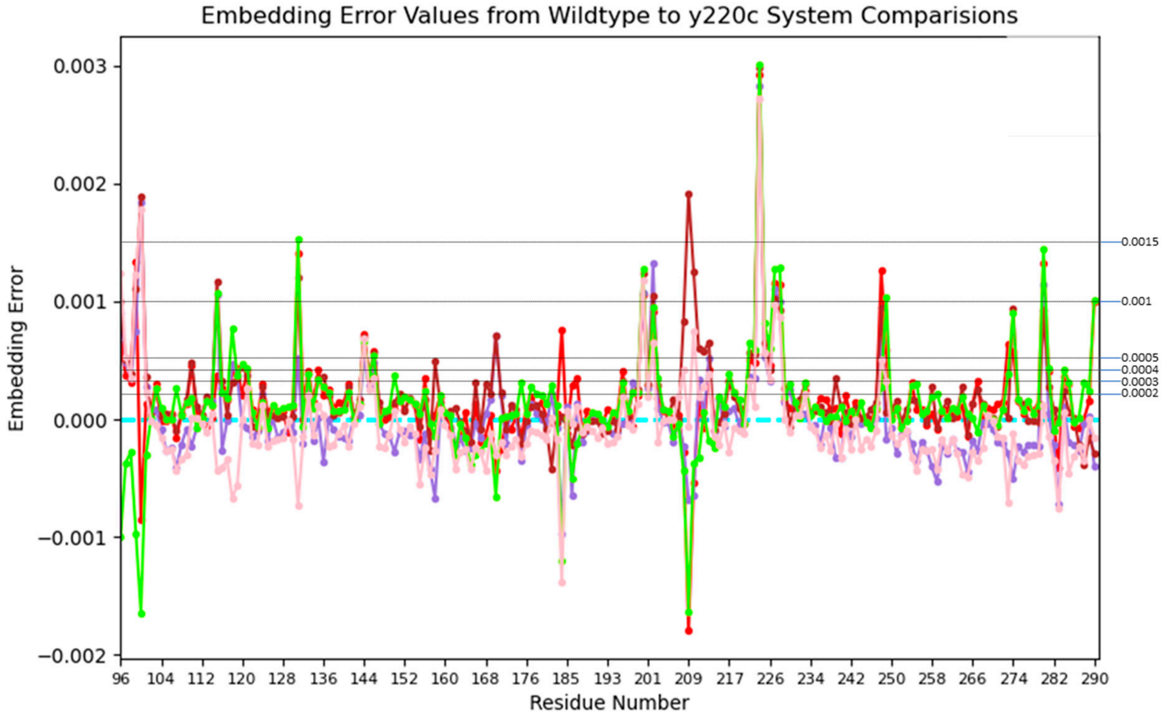

Supplementary Figure S5. EED distributions of Figure 4 with positive EED ranges corresponding to increasing EED values greater than or equal to 0.0002, 0.00025, 0.0003, 0.0004, 0.0005, 0.001, and 0.0015 labeled and indicated by dotted lines. These ranges of positive EED value were used to investigate the regions across the DBD displaying "significant" rescue as projected on DBD structure in Figure 5 and displayed in Table 1. X-axis corresponds to residue index and Y-axis EED value calculated as the difference (error) between each residues' EE value from the y220c-ub_y220c-j and wt_y220c-j distributions where j is one of the five examined drug compounds: 8, 20, 22, 27, pk11000. A blue dotted line is drawn across the x-axis to indicate an EED value of 0. Secondary structural elements of the p53-DBD are visualized under the x-axis. Coloration as dependent on construct y220c-j follows from Figure 2.

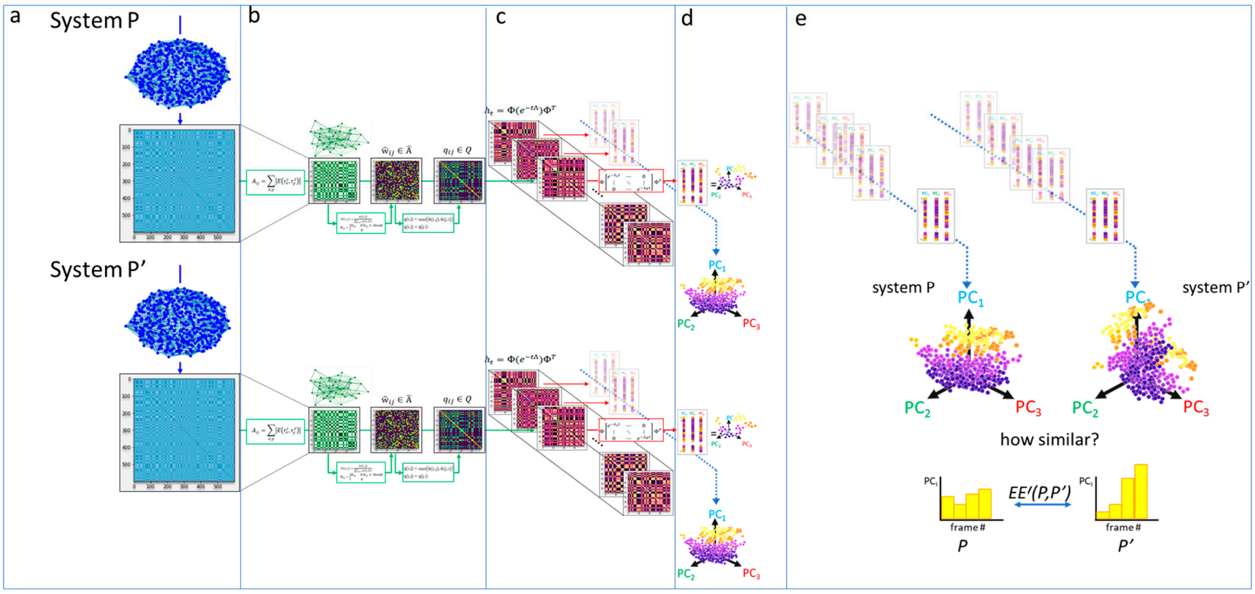

Supplementary Figure S6. Flowchart diagram indicating processing of Electrostatic Networks into EED distributions for two constructs P and P'. (a) Raw electrostatic atom-wise networks for both systems P and P'. (b)Conversion of atom-wise electrostatic networks into residue-wise networks and subsequent processing through local thresholding procedure. (c) Generation of heat kernel for each frame of electrostatic network. (d) conversion of each frame's generated heat kernel into the latent R3 PC space. (e) Computation embedding error (EE) value between each PC. Calculation of EE value is visualized for PC1 here for simplicity.
