# Supplementary material for: Network Theory Analysis of Allosteric Drug-Rescue Mechanisms in the Tumor Suppressor Protein p53 Y220C Mutant
Source: Int J Mol Sci. 2025 Jul 17;26(14):6884. doi: 10.3390/ijms26146884 (PMC12295982; doi:10.3390/ijms26146884)
Supplement: Supplementary file 1 [file ijms-26-06884-s001.zip › Supplementary_Material_Method Calculations.pdf]

Supplementary Material on Electrostatic Network and Heat Kernel Time Parameter Metrics:

Benjamin S. Cowan[1] and Kelly M. Thayer[1,2,3*]

[1,2]Department of Computer Science
Wesleyan University
Middletown, CT 06457 USA
[2]Molecular Biophysics Program,
Wesleyan University
Middletown, CT 06457 USA
[2]Department of Chemistry,
Wesleyan University
Middletown, CT 06457 USA

Locally Thresholded and Normalized Energetic Network Generation Protocol.

In our analysis, we want to capture residue-residue levels of energetic interaction with an atomic level of resolution for subsequently embedding the protein as a network structure of its constituent residues' energetic interactions. Through the python package *pytraj*, we follow Abramson's implementation of a simple algorithm to convert these atom-wise energetic interactions into residue-wise energetic interactions [1]. In brief, this algorithm parses the topology file of the protein construct and maps into a dictionary each atom in the trajectory file to an associated residue index. Once the atom-residue mapping is generated, a pairwise matrix is created from the original atom-atom pairwise interaction matrix wherein each matrix entry holds the accumulated sum total of all atom-wise interaction energy between every pair of residues [1]. As such, for a p53-DBD construct, each frame's 2999x2999 pairwise atom-atom energetic interaction matrices are mapped into a 193x193 residue-residue energetic interaction matrix. Each matrix of a frame therefore represents a weighted adjacency matrix where the nodes represent the protein's composite residues and edge weights represents the level of energetic interaction (either electrostatic or Van der Waals) between each residue pair at a particular instance of simulation time. This weighted adjacency matrix A is given by:

$$A_{ij} = \sum_{x,y} \left| E\left(r_x^i, r_y^j\right) \right| \tag{1}$$

where i,j is a pair of residues, x,y are atoms in residues i and j respectively, and $E(x, y)$ gives the pairwise atomic-interaction energy computed by the pairwise command [1]. As such the sum of these atomic energy values for residues i and j gives us the associated pairwise energetic interaction value between them. This interaction energy value represents the edge weight (i,j) in the weighted adjacency matrix between nodes i and j.

Once we have calculated these 193x193 residue interaction networks for each of the two energy channels over all 152 sampled frames of a p53-DBD construct's MD trajectory, they are parsed into a 152x2x193x193 data tensor. Tensors are a multi-dimensional nested array datatype which act as an efficient data structure for storing high-dimensional data (such as a time series of graph structures) and computing operations over accessed vectors within them in an organized manner.

While these energetic networks embed edge weight values by the level of energetic interaction between each residue pair, the embeddings themselves don't sufficiently account for the impact of how the local energetic environment of each residue effects each of its own pairwise interactions. As such, the capacity of each residue to energetically interact with other residues is relative to how energetically "ordered" the interactions of its surrounding energetic environment are. Residues in more highly ordered regions of a protein tend to experience a higher net interaction force to maintain a certain level of energetic and structural stability compared to those in more disordered and or flexible regions of the protein [2]. As such, each frame's energetic network for each energy channel undergoes a normalization and thresholding protocol to account for such local energetic-interaction discrepancies acting on each residue. The normalization component of this process is performed by calculating what is referred to as the localized interaction matrix $\hat{A}_{\mathrm{nxn}}$. For every pairwise edge weight value *w(i,j)* between residues i and j in the weighted adjacency matrix A, each locally normalized edge value in $\hat{A}$,$\hat{\mathrm{w}}(i,j)$ between residues i and j is given by:

$$\hat{\mathrm{w}}(i,j) = \frac{\mathrm{w(i,j)}}{\sum_{k=1}^{n} w(i,k)} \tag{2}$$

By calculating the normalized interaction matrix, each edge of the energetic graph is converted to the proportion of total interaction energy experienced by a corresponding node. For example, let G = (V, E) complete weighted undirected graph where V= {u, v, x, y, z} and the corresponding pairwise energetic interaction matrix of G represents each edge weight between every node pair (i, j). For the unnormalized edge (u,v), node *u*'s energetic interaction with node *v* represented by weight value *w(u,v)* with node *u*'s remaining unnormalized edge weight values in the interaction matrix being *w(p, x) = 5*, *w(p, y) = 6,* and *w(p, z) = 2*, the locally normalized edge weight $\hat{\mathrm{w}}(\mathrm{u},\mathrm{v}) = \frac{5}{5+6+2} = 0.38$. Additionally, this process effectively sparsifies the network, pruning edges where the local energetic environment less effects a residue's energetic interactions while emphasizing regions in the network where such environmental energetic contributions significantly occur [1].

Since this procedure for normalizing edge weight values respective to each node's environmental energetic interactions results in different normalization weights for an edge dependent on which node it is calculated relative to, the graph becomes directed. Thus, in order to ensure graph undirectedness and symmetry, for directed edges *(u,v)* and *(v,u)* between each pair of nodes u and v in a graph, if the value of ŵ(u,v) is greater than ŵ(v,u), ŵ(u,v) is said to have a greater "influence" on the local energetic environment it describes [2]. As such, we calculate from the locally normalized energetic interaction matrix an *influence matrix* [1] by determining greater of the normalized energetic interaction edge weights ŵ(u,v) and ŵ(v,u) between each pair of nodes *u* and *v* and setting both edge weight to the greater of these two weight values. Moreover, in order to increase the signal to noise ratio of the resultant energetic influence matrix and further increase graph sparsity and numerical stability, we threshold all edge weights to 0 with a weight less than a specified cutoff value. In our analyses, this cutoff value was set to 0.003 to include the majority of the energetic network's edge weight values while cutting out excessively low and minimal residue energetic interactions that could introduce noise in subsequent steps of heat kernel generation and projection.

Optimization Protocol for Determining Heat Kernel Time Parameter

As calculation of the heat kernel and the embedded representation of the network topology which it describes is dependent on the time parameter *t* (i.e., the amount of time we diffuse heat across the network), we must determine the appropriate value of this parameter such that we can best describe the majority of the variance in the network topology as embedded by the heat kernel within a lower dimensional eigenvector space. To achieve and partially automate this process, we utilize an implementation [3] of a "knee point" detection algorithm based upon calculating the point of maximum curvature for discrete datasets [4]. When working with data, it is sometimes important to know where a data point's "relative costs to increase some tunable parameter is no longer worth the corresponding performance benefit" [4]. In our analyses, this "tunable parameter" is the number of principal components necessary to capture the majority of variance embedded by the heat kernel. As a principal component's eigenvalue represents the "importance" of that principal component vector for capturing the numerical variance of a matrix, the specific eigenvalue in an eigenvalue distribution *beyond which* subsequent eigenvalues tend to "level" off in value (and thus add little performance benefit in describing the variance of the matrix) is the "knee point" we are interested in determining. While normally such a conception of a knee point is encapsulated by computing the point of maximum curvature in a continuous distribution, for matrices, such as the heat kernel matrix consist of discrete vectors, determining the inflection point as a continuous value for an eigenvalue distribution is not meaningful. The authors of the 'kneedle' algorithm address this problem by quantifying the point(s) of maximum curvature in a discrete dataset as approximately the set of points in a curve that are local maxima if the curve is rotated θ degrees clockwise about (x_{min}, y_{min}) through the line formed by the points (x_{min}, y_{min}) and (x_{max}, y_{max}). After rotating a discrete dataset about this line, the local maxima— and thus knees—are the points at which the curve differs most from the straight line segment connecting the first and last data point of the distribution, thereby *approximating* the point of maximum curvature for a discrete set of points. We briefly describe this algorithm as follows:

Given a distribution of points $D = \{(x_i , y_i) \in R^2\}$:

1. The algorithm first transforms D into a new dataset D_s where the original (x_i,y_i) values are fitted to a smoothing spline in order to preserve the original shape of the distribution:

$$D_s = \{(x_{si}, y_{si}) \in R^2 | x_{si}, y_{si} \geq 0\} \quad (3)$$

2. To normalize the magnitude of datapoints in D_s, the set D_{sn} is created, containing the datapoints (x_{si}, y_{si}) normalized to the unit square as (x_{sn_i}, y_{sn_i}):

$$D_{sn} = \{(x_{sn_i}, y_{sn_i}) \in R^2 | (x_{si}, y_{si}) \in D_s\} \quad (4)$$

3. A new set is created, D_d, which represents the set of differences between the x and y values of D_d to determine when the curve changes from horizontal to sharply increasing (or decreasing) indicating the presence of a knee in the original dataset:

$$D_d = \{(x_{di}, y_{di})\} \text{ where: } \quad x_{d_i} = x_{sn_i} \quad y_{d_i} = y_{sn_i} - y_{sn_i} \quad (5)$$

4. To find the knee points of the normalized curve described by D_s, the set of local maxima points: $D_{\mathrm{lmx}} = \{(x_{\mathrm{lmx},i}, y_{\mathrm{lmx,i}})\}$ of the difference curve as described by D_d are computed (e.g., the datapoints where the difference curve flattens out).

5. To determine which of these local maxima is the actual knee of the difference curve, a unique threshold value is defined for each local maximum, $T_{lmx,i}$ based on the average difference between consecutive x-values and a sensitivity parameter, S, which measures how many flat points we expect to see in the data curve before (or after) declaring a knee.

$$T_{lmx,i} = y_{lmx,i} - S \cdot \frac{\sum_{i=1}^{n-1}(x_{sn_{i+1}} - x_{sn_i})}{n - 1} \qquad (6)$$

6. To determine the knee of the original distribution:
   - If any difference value (x_{dj} , y_{dj}) exists where j > i drops below the threshold $y = T_{lmx,i}$ for ($x_{lmx,i}$, $y_{lmx,i}$) before the next local maximum is reached, kneedle declares a knee at the x-value corresponding to $x = x_{lmx,i}$.
   - If the difference values reach a local minimum and starts to increase before $y = T_{lmx,i}$ is reached, the threshold value is reset to 0 until another local maximum is reached.

For a distribution D of *n*-pairs of x and y values, the time complexity of the kneedle algorithm to determine the knee point is bounded by $O(n^2)$ [4].

Utilizing this algorithm enables us to determine the appropriate number of principle components necessary to retain for capturing the numerical variance of the mean centered heat kernel for various diffusion times. The mean-centered heat kernel is simply computed as the average heat kernel of a p53-DBD construct across all 152 frames of the MD simulation trajectory.

In our protocol, we seek towards determining the optimal value of the time parameter that captures the majority of the mean-centered heat kernel's numerical variance within the first three leading principal component's eigenvalues. The decision to use the leading three principle components as this cutoff metric for determining the time parameter was inspired by prior studies which utilized the leading three principle components to characterize graph-structural properties of geometric manifolds via a latent heat kernel embedding procedure [5]. The reference means centered heat kernel which we use to determine the time parameter to use for all p53-DBD constructs is that generated from the wildtype p53-DBD trajectory which we rationalize as capturing a "baseline" of the most important variances of p53-DBD network dynamics.

1. Abramson, D. I. The Colors of a Protein: Protein Dynamics Through the Lens of Spectral Graph Theory. Honors Thesis, Wesleyan University, Middletown, Connecticut, 2021.
2. Cowan, B., Interconnected Protein Networks: Insights Towards CRIB-Par6 Protein Allostery Through a Graph-Theoretic Analysis. **2022**.
3. Arvai, K. *Kneed: Knee-point detection in Python*, GitHub repository, 2020.
4. Satopaa, V.; Albrecht, J.; Irwin, D.; Raghavan, B. In *Finding a "Kneedle" in a Haystack: Detecting Knee Points in System Behavior*, 2011 31st International Conference on Distributed Computing Systems Workshops, 20-24 June 2011; 2011; pp 166-171.

5. ElGhawalby, H.; Hancock, E. R., Heat Kernel Embeddings, Differential Geometry and Graph Structure. **2015,** *4* (3), 275-293.
